# A call for broadening the altmetrics tent to democratize science outreach

**DOI:** 10.1371/journal.pbio.3003010

**Published:** 2025-02-07

**Authors:** Ivan Jarić, Pavel Pipek, Ana Novoa

**Affiliations:** 1 Université Paris-Saclay, CNRS, AgroParisTech, Gif-sur-Yvette, France; 2 Biology Centre of the Czech Academy of Sciences, Institute of Hydrobiology, České Budějovice, Czech Republic; 3 Department of Invasion Ecology, Institute of Botany, Czech Academy of Sciences, Průhonice, Czech Republic; 4 Department of Ecology, Faculty of Science, Charles University, Prague, Czech Republic; 5 Estación Experimental de Zonas Áridas, Consejo Superior de Investigaciones Científícas (EEZA-CSIC), Almería, Spain

## Abstract

Common altmetrics indices are limited and biased in the social media that they cover. This Perspective highlights how and why altmetrics should broaden its scope to provide more reliable metrics for scientific content and communication.

## Teaser

Common altmetrics indices are limited and biased in the social media that they cover. In this Perspective, we highlight how and why altmetrics should broaden its scope to provide more reliable metrics for scientific content and communication.

The need to measure and understand societal impact of research, together with the widespread use of online platforms to disseminate scientific content, has led to efforts to develop indicators of scholarly work uptake beyond traditional scientometrics. These metrics, known as altmetrics, represent web-based measures of societal attention and engagement with scientific publications. Altmetrics provide a complementary measure of research impact by tracking diverse online sources, such as social media, blogs, and news articles, to capture the broader societal uptake of research outputs [1].

**Fig 1:**
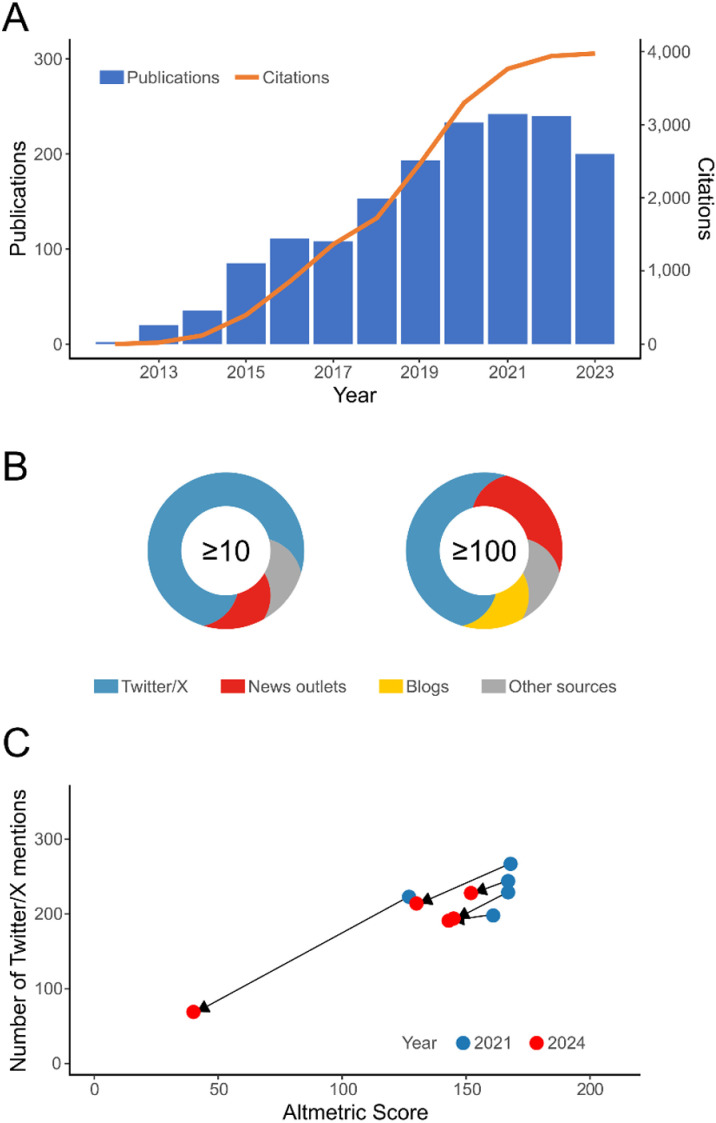
Impact of research on altmetrics, and the structure and temporal instability of Altmetric scores. **(A)** Publication output and impact of research related to altmetrics, based on Web of Science Core Collection search using the query “altmetric*” to search within topics. **(B)** Average proportion of Altmetric scores for papers published in *PLOS Biology* during 2021–2024 representing different online media sources. Left and right Altmetric badges represent publications with Altmetric scores of at least 10 and 100, respectively. **(C)** Temporal decline in Altmetric scores and X/Twitter mentions between November 2021 and November 2024 (i.e., following Twitter acquisition by Elon Musk in 2022 and US presidential elections in 2024) of the top five most mentioned papers published in the journal Biological Invasions.

Various platforms providing scientific article-level altmetrics have been developed by different companies and organizations, such as Altmetric (Altmetric.com), Plum Analytics (PlumX), Crossref (Event Data), and OurResearch (ImpactStory), while several publishers provide their own sets of altmetrics indices for their journals. Altmetrics have been already implemented in some major scientific databases [2], and research institutions and scientific journals use them to assess their performance [3]. Altmetrics are receiving increasing scientific attention (Fig 1A), and they are being used in job and grant evaluations, as well as for academic rankings [4,5]. The importance of the information collected by altmetrics is likely to be further strengthened with the wider adoption of the Declaration on Research Assessment (DORA), launched in 2012 with the aim of moving away from the use of the impact factor by introducing a wider range of evaluation criteria (https://sfdora.org/read/).

Despite their growing influence and value, altmetrics have significant limitations. First, stakeholders (e.g., online news and other media content creators) are not always aware of the relevance of altmetrics and of how they work—for example, scientific papers often feature on online platforms without their DOI, and such posts do not get recognized by altmetric platforms. Effective tracking of scholarly impact would require improved stakeholder awareness and cooperation.

Second, altmetrics are often presented as a single composite score of societal impact, or at best as a set of a few metrics. However, the way such scores are calculated is not fully transparent, and it is consequently challenging to interpret them meaningfully [6]. The greatest importance of altmetrics should rather lie in highlighting the actual content and context featuring the public engagement with scientific outputs [6], considering that high altmetrics scores can also be generated by negative reception of the results by the community. This could be achieved automatically, for example, with the use of Large Language Models [7], such as ChatGPT or Claude, which can interpret texts in a contextually meaningful way and extract topics and sentiments associated with the altmetrics scores.

Third, most altmetrics platforms only consider a narrow selection of mainstream media sources. This is especially true for social media coverage. For instance, the prominent altmetrics platform PlumX currently collects data exclusively from Facebook, while Altmetric, until very recently, has tracked just three additional major social media platforms—X (formerly Twitter), Reddit and Youtube. However, many other social networking services are not covered, including open-source and decentralized platforms such as Mastodon. Additionally, they are heavily biased towards English content, with the dissemination through non-English communications (such as large social media platforms in China and India, often with hundreds of millions of users) being particularly neglected [8]. While we acknowledge the challenges linked to the issue of state censorship in some of those platforms [9], such selective focus raises concerns about the inclusivity, accuracy and usefulness of altmetrics scores. By ignoring online communication and outreach activities beyond a few selected media platforms, altmetrics are effectively failing to capture the full spectrum of scholarly communication and true scholarly impact, and underrepresenting research disseminated through alternative media platforms.

Biases in social media coverage by major altmetrics platforms can lead to a significant monopolization of the social media landscape, by inadvertently making scholars feel pressured to confine their dissemination and communication efforts to a handful of platforms recognized by altmetrics systems, to ensure that their research impact will be properly acknowledged. This is especially true considering that dissemination activities and discussions on social media tend to represent the major part of the altmetrics scores (Fig 1B). Growing concerns about platforms like X, due to issues of content moderation and disinformation [10], further highlight the urgency of enhancing the altmetrics coverage. Following the recent platform takeover by Elon Musk, there has been a surge in migration of scientists from X to other platforms such as Mastodon and Bluesky, which was further intensified following the 2024 US presidential elections [11].

We acknowledge that broadening the coverage of social media platforms is not an easy task, but we are positive that this might happen in the future. For example, Bluesky has been recently included in Altmetric scoring, and it remains to be seen whether other altmetrics platforms such as PlumX will follow suit. However, this problem needs to be addressed in a more systematic way, by accounting for a wider range of online media sources, and especially by a more balanced regional and linguistic coverage.

Finally, the ongoing migration from X to other social media platforms points to another weakness of altmetrics: their temporal instability [5]. When an X account gets deactivated, all the posts that have mentioned scientific publications cease to be accessible, which automatically leads to a reduction of altmetrics scores (Fig 1C). Altmetrics scores can also be affected by online platforms being closed or banned, or by changes in altmetrics companies’ coverage of online sources, as for example happened when PlumX ceased tracking X in 2023 or when Altmetric ceased following Pinterest (in 2013), LinkedIn (in 2014) or Weibo (in 2015). To address the issue of temporal instability, altmetrics scores should always include a timestamp. Furthermore, it would be important to ensure adequate level of interoperability among social media platforms, allowing users to transfer the content between platforms [12]. Such measures would help maintain social media content when users switch between platforms, as well as help diminish present monopolization of the social media landscape. Furthermore, wider adoption of common protocols, such as ActivityPub in Fediverse, or of bridging services, such as Bridgy Fed, would allow greater visibility, communication and data sharing among media platforms.

The issue is overall quite complex, and our aim is not to cover exhaustively all the relevant aspects. Our intention is to initiate a constructive discussion that should involve the scientific community and key stakeholders. We believe that, to support a more inclusive and democratic picture of scholarly impact, altmetrics platforms should broaden their scope to include a wider array of social media platforms and more non-English content, address the temporal instability of altmetrics through timestamping, and be more transparent about how they track and quantify public attention. This together would allow for a better capture of the global reach and societal impact of research, and support a more democratic and diverse scientific discourse.
